# Fabrication of Nanostructured Polycaprolactone (PCL) Film Using a Thermal Imprinting Technique and Assessment of Antibacterial Function for Its Application

**DOI:** 10.3390/polym14245527

**Published:** 2022-12-16

**Authors:** Hee-Kyeong Kim, Se-Jin Jang, Young-Sam Cho, Hyun-Ha Park

**Affiliations:** 1 Department of Mechanical Engineering, College of Engineering, Wonkwang University, 460 Iksandae-ro, Iksan 54538, Republic of Korea; 2 Department of Mechanical Design Engineering, College of Engineering, Wonkwang University, 460 Iksandae-ro, Iksan 54538, Republic of Korea; 3 MECHABIO Group, Wonkwang University, 460 Iksandae-ro, Iksan 54538, Republic of Korea

**Keywords:** antibacterial, nanostructure, polycaprolactone, flexible film

## Abstract

In the use of the medical devices, it is essential to prevent the attachment of bacteria to the device surface or to kill the attached bacteria. To kill bacteria, many researchers have used antibiotics or studied nanostructure-based antibacterial surfaces, which rely on mechanical antibacterial methods. Several polymers are widely used for device fabrication, one of which is polycaprolactone (PCL). PCL is biocompatible, biodegradable, easy to fabricate using 3D printing, relatively inexpensive and its quality is easily controlled; therefore, there are various approaches to its use in bio-applications. In addition, it is an FDA-approved material, so it is often used as an implantable material in the human body. However, PCL has no inherent antibacterial function, so it is necessary to develop antibacterial functions in scaffold or film-based PCL medical devices. In this study, process parameters for nanopillar fabrication were established through a simple thermal imprinting method with PCL. Finally, a PCL film with a flexible and transparent nanopillar structure was produced, and the mechano-bactericidal potential was demonstrated using only one PCL material. PCL with nanopillars showed bactericidal ability against *Escherichia coli* (*E. coli*) and *Bacillus subtilis* (*B. subtilis*) bacteria cultured on its surface that resulted in membrane damage and death due to contact with nanopillars. Additionally, bacteriostatic results were shown to inhibit bacterial growth and activity of *Staphylococcus aureus* (*S. aureus*) on PCL nanostructured columns. The fabricated nanopillar structure has confirmed that mechanically induced antibacterial function and can be applied to implantable medical devices.

## 1. Introduction

Membrane-type or three-dimensional structure-type medical devices need to be made of biocompatible materials, which can be metal, bioceramic, or polymer [1,2,3]. Biocompatible polymers can be categorized as follows: natural polymers and synthetic biocompatible polymers. Synthetic polymers include polylactic acid (PLA), polyglycolic acid (PGA), and polycaprolactone (PCL), which are inexpensive and easy to manufacture [4,5,6]. Moreover, they have relatively good mechanical strength [7]. In particular, PCL has relatively good elongation at break and high flexibility, and as an FDA-approved material, it is a promising material for clinical usage [2,8,9]. However, inserting a membrane or scaffold based on PCL into the body may cause an inflammatory reaction or infection caused by bacteria [10]. To solve this problem, researchers have applied nanotubes [11,12,13], graphene [12,14,15,16], silver [17,18,19,20], metallic ion [21], and peptides [22,23,24] to the PCL surface. However, newly proposed biomaterials and nanomaterials require nonclinical information to be obtained, and commercialization is difficult due to strict FDA clinical regulation. In addition, to kill bacteria, a surface should damage the bacterial membrane using the nanostructure [25,26,27]. Meanwhile, several studies have reported mechanically induced bactericidal activity on implanted metallic devices [25,28,29,30]. However, there are very few studies of nanofabrication based on PCL, and the reported studies do not focus on the antibacterial effects but on cell proliferation and differentiation [31,32,33,34].

In this study, we investigate a mechano-bactericidal method based on nanostructures as an antibacterial method. In the case of the mechano-bactericidal method, only the characteristics of the material are used to damage the bacteria membrane attached by the nanostructure [35]. In addition, nanopillars have higher mechanical strength than our previously reported nanocone shapes, and the antibacterial efficiency of nanopillars has also been verified [36,37,38,39]. Therefore, nanopillars were fabricated through a thermal imprinting method based on FDA-approved PCL. We found that the temperature at which it is demolded affects the shape of the nanopillars. Through a demolding temperature optimization experiment, we confirmed the optimum temperature at which the shape of the nanopillar was well-fabricated over a large area. The fabricated nanopillared array is flexible and transparent and can be applied to curved surfaces. Three types of bacteria were cultured on the prepared surface, and antibacterial effects were confirmed on the prepared nanopillared surface. This overcomes the problems seen in existing PCL-based antibacterial surfaces. This PCL-based nanopillared surface can be applied as an important element to solving the problems that occur when a device is inserted into the human body in tissue engineering and regenerative medicine applications.

## 2. Materials and Methods

### 2.1. Materials

Polycaprolactone (PCL) (M_w_: 45,000), dichloromethane and trichloro(1H,1H,2H,2H-perfluorooctyl) silane were purchased from Sigma-Aldrich (St. Louis, MO, USA). Polyurethane acrylate (PUA) and polyethylene terephthalate (PET) were purchased from Minuta Tech (311 RM; Minuta Tech, Osan, Republic of Korea).

### 2.2. Fabrication of the PCL-Based Nanopillars

A Si master with nanopillars was treated by trichloro(1H,1H,2H,2H-perfluorooctyl) silane solution for easy demolding. The PUA was dropped onto the Si master and covered with a PET film. To remove the bubbles, the PUA sample was degassed for 3 h in a vacuum chamber. After the degassing process, the PUA sample was exposed to ultraviolet (UV) light (λ = 365 nm, dose = 200 mJ/cm^2^) for photopolymerization, and the cured PUA film was carefully removed from the Si master. The prepared PUA sample was treated with a trichloro(1H,1H,2H,2H-perfluorooctyl) silane solution for fluorination.

**Figure 1:**
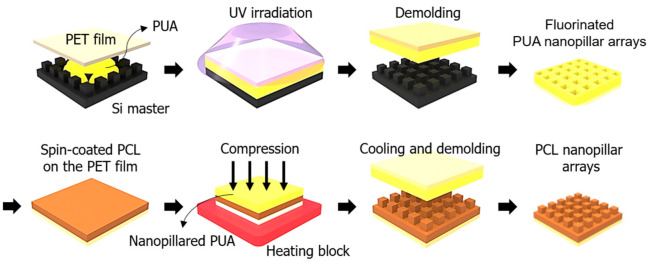
Schematic of the fabrication process for PCL nanopillar arrays using the thermal imprinting technique.

The PCL was dissolved in dichloromethane by stirring for 3 h with a magnetic bar to obtain an 18 wt% concentration of PCL solution [31]. To fabricate the nanopillar, the PCL solution was spin coated onto 100 μm-thick polyethylene terephthalate (PET) at 3000 rpm for 30 s. The treated PUA sample was placed on the PCL surface and embossed with a pressure of 15 MPa at 80 °C for 1 min with a hot embossing machine (TD-HP01, TnDorf, Bucheon, Republic of Korea). The PUA and PCL were formed to replicate the nanopillar. Then, the PUA and PCL samples were cooled for 30 min at different temperatures (0, 10, 20, 30, 40 °C), and the PUA sample was peeled off from the PCL layer (Figure 1). The bare (flat) PCL of the control group was fabricated under same conditions as the nanopillared PCL sample using 18 wt% PCL solution. It was fabricated using a silicon wafer instead of the PUA nanopillar mold to make the flat surface of bare PCL. The resulting fabricated PCL film with nanopillars was flexible and transparent. For the biological experiments, the PCL film was sterilized by 70% ethanol and UV exposure for 1 h.

### 2.3. Scanning Electron Microscopy (SEM)

The PCL nanopillared surface was coated using metal sputtering (G20, GSEM, Suwon, Republic of Korea) with a 5 nm thickness of platinum (Pt) to avoid charging. Then, the coated surface was analyzed by scanning electron microscopy (SEM: SU8200 microscope, Hitachi, Chiyoda Ward, Japan).

### 2.4. FT-IR Spectroscopy Analysis

Fourier transform infrared spectroscopy (FT-IR) was performed with a 6300FV + IRT5000 (JASCO, Tokyo, Japan) using a horizontal germanium crystal attenuated total reflection (ATR) plate.

### 2.5. Measurements of Water Contact Angle and Optical Transmittance

To observe the hydrophobicity of the fabricated PCL nanopillared surface, the water contact angle (WCA) was measured by a drop angle analyzer (SMARTDROP_PLUS_HS, FEMTOFAB, Pohang, Republic of Korea). The drop volume of distilled water was 5 μL, which was dropped onto fabricated PCL samples at room temperature (relative humidity 45%, 25 °C). The average values of the water contact angle were obtained from five repeated measurements at random positions. The optical transmittance of the PCL nanopillared sample was measured by a UV-Vis-NIR spectrophotometer (UH4150, Hitachi, Chiyoda Ward, Japan) in the 300–900 nm wavelength range.

### 2.6. Antibacterial Tests

The mechano-bactericidal method relies on penetration and stretching mechanisms, and the bacteria membrane is damaged by both mechanisms. Herein, the nonactivation of bacteria is attributed to a peptidoglycan layer in the bacteria [40,41]. According to the hypothesis, the bacteria membrane can be damaged by both mechanisms. Because of the abovementioned mechanisms, the thickness of bacteria membrane is one of the important properties for assessing the results of mechano-bactericidal experiments. In general, bacteria can be categorized into Gram-negative and Gram-positive types. One of distinguishable characteristics is the thickness of the bacteria membrane, which is dependent on the thickness of the peptidoglycan layer. The motility of bacteria is an additional important parameter in assessing results of mechano-bactericidal experiments because the movement of bacteria can affect the damage of the bacteria membrane on the nanostructured surface. Consequently, in general, most researchers in this field select three types of bacteria for testing in mechano-bactericidal experiments: *Escherichia coli* (ATCC 25404), a Gram-negative and motile type; *Bacillus subtilis* (ATCC 21332), a Gram-positive and motile type; and *Staphylococcus aureus* (ATCC 25923), a gram-positive and nonmotile. The bacteria were grown in Luria broth (LB Broth Miller, BD Difco, Franklin Lakes, NJ, USA) within a shaking incubator at 37 °C and 170 rpm until an optical density of 0.3 at 600 nm (OD_600_) was reached. The bacterial suspension was diluted to an OD_600_ of 0.1 (*E. coli*, 1 × 10^6^ CFU/mL; *B. subtilis*, 2 × 10^6^ CFU/mL; and *S. aureus*, 2 × 10^7^ CFU/mL) and incubated on the sterilized PCL samples at 37 °C for 18 h. To observe the live/dead viability of bacteria, the attached bacteria on the samples were stained with Live/Dead BacLight bacterial viability kit (BacLight™, L7012; Molecular Probes, Invitrogen, Carlsbad, CA, USA) for 15 min in the dark, and the stained PCL samples were then rinsed with phosphate-buffered saline (PBS). Bacteria live/dead staining was performed according to the BacLight bacterial viability kit protocol. The stained bacteria on the PCL samples were analyzed using a fluorescence microscope (LSM 980; Carl Zeiss, Jena, Germany). Additionally, the prepared PCL samples were incubated in a bacterial suspension of 2 mL (OD_600_ = 0.1) for 18 h at 37 °C for a colony forming unit (CFU) assay and carefully washed with PBS. The rinsed samples were transferred into a conical tube with 1 mL PBS and cleaned using ultrasonic cleaner (40 Khz, NXP-1002, kodo, Hwaseong, Republic of Korea) for 2 min to detach the bacteria from the samples. After serial dilution, the bacterial solution was spread on LB agar plates and incubated for 18 h at 37 °C. Lastly, the grown bacterial colonies on the agar plates were counted (the antibacterial test was performed using three specimens for each condition). To evaluate the antibacterial ability, the antibacterial rate was calculated via Equation (1):

(1) $$\mathrm{Antibacterial}\;\mathrm{rate}\;\left(\%\right)=\frac{{\mathrm{CFU}}_{\mathrm{control}}-{\mathrm{CFU}}_{\;\mathrm{experimental}\;}}{{\mathrm{CFU}}_{\mathrm{control}}}\times 100$$

where CFU_control_ is the average number of CFU on the bare PCL, and CFU_experimental_ is the CFU of bacteria on the nanopillared PCL.

After the antibacterial test, the bacteria attached to the PCL sample surface were observed through SEM. After incubation, the PCL samples were gently washed using PBS and fixed using 4% paraformaldehyde in PBS (GeneAll, Seoul, Republic of Korea) for 15 min at room temperature. The fixed samples were washed five times. The samples were then transferred to a new plate and dehydrated through a series of ethanol solutions of 20, 40, 60, 80, and 100 vol% for 15 min, respectively. Before observation, samples with attached bacteria were completely dried and then coated with platinum (5 nm) using a sputtering coater (E-1045, Hitachi, Chiyoda Ward, Japan).

To quantify the biofilm biomass, we used the crystal violet (CV, Sigma-Aldrich, St. Louis, MO, USA) assay [42]. Before the CV staining, cultured PCL samples (1 × 1 cm^2^) for 18 h were gently washed using PBS. The biofilms were then stained with 0.1% CV solution for 20 min. Each sample was washed twice with D.I. water, and the bound CV was released with 95% ethanol. To estimate the total biofilm biomass, the OD of the resulting solution was measured at 600 nm. The biofilm biomass assay was performed using five specimens.

## 3. Results and Discussion

### 3.1. Surface Characterization of the PCL Nanopillar Arrays by Demolding Temperature

**Figure 2:**
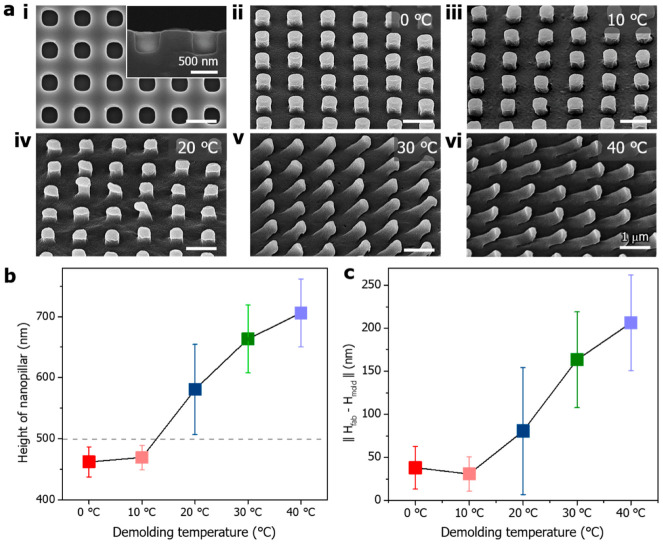
(**a**) SEM images: (**i**) negative-type PUA nanopillar mold surface; PCL nanopillars detached from the mold at (**ii**) 0 °C, (**iii**) 10 °C, (**iv**) 20 °C, (**v**) 30 °C, and (**vi**) 40 °C; (**b**) height of the nanopillar with respect to the demolding temperature; and (**c**) height difference between the mold and fabricated nanopillar with respect to the demolding temperature.

The negative-type PUA nanopillar surface was formed by a UV molding process and has a precise nanopillar (Figure 2(ai)). The individual nanopillars in the PUA mold had a depth of 500 nm, spacing of 500 nm, and a diameter of 500 nm. The fabricated PCL nanopillar shapes were affected by the demolding temperature. As seen in the SEM images from Figure 2(aii) to Figure 2(avi), the PCL nanopillar stretched as the demolding temperature increased. When removed from the PUA mold at 0 °C (Figure 2(aii)), the PCL nanopillars show good morphology. The fabricated shape was realized with a diameter of 500 nm, spacing of 500 nm, and a height of approximately 490 nm, similar to the size of the PUA mold. The PCL nanopillars detached at 10 °C (Figure 2(aiii)) and 20 °C (Figure 2(aiv)) showed slight deformation. In particular, Figure 2(av) and Figure 2(avi) show damaged nanopillars at 30 °C and 40 °C, respectively. They were stretched and slightly bent. Therefore, it can be concluded that the best results are obtained at 0 °C. The height of the nanopillars was measured according to the demolding temperature, and Figure 2b shows that the pillar height increases as the removal temperature increases. Additionally, as the demolding temperature increased, the difference between the depth of the PUA mold nanopillar and the height of the fabricated PCL nanopillars increased (Figure 2c). When the demolding temperature was lower, the releasing force and the stretching tension at the interface between the mold and the replica seem to be reduced [43], and the deformation of the nanopillars was negligible at a low temperature. However, as the release temperature increased, the mechanical properties, yield strength or stiffness could decrease because the melting temperature of PCL was approximately 60 °C [44,45]. Therefore, the PCL nanopillars were more elongated as the temperature increased. In addition, it was found that the orientation of the nanopillars was also determined by the release direction.

**Figure 3:**
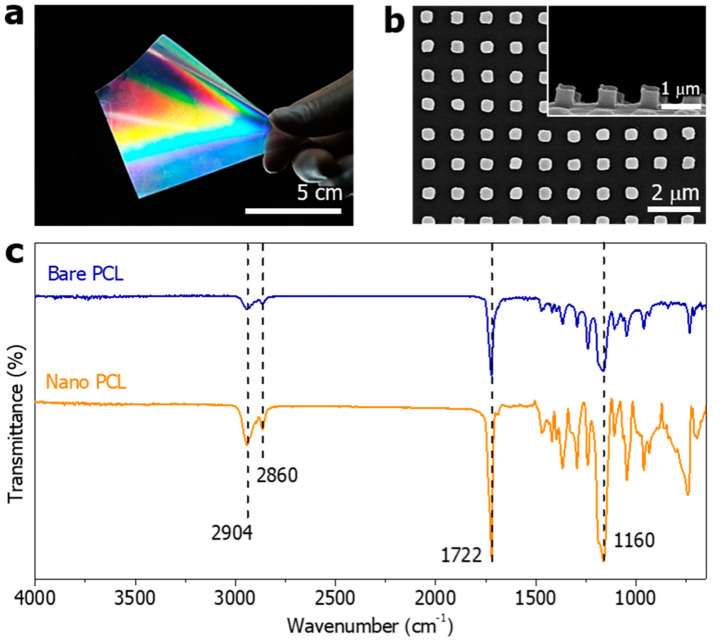
(**a**) Photograph of the flexible PCL film with nanopillar arrays. (**b**) SEM images of the PCL nanopillar arrays at 0 °C demolding temperature. (**c**) FT-IR spectrum of the bare PCL and nanopillared PCL.

When the demolding temperature was 0 °C, the shape of the nanopillar was uniformly formed in a large area. The final product is a transparent and flexible film with well-formed nanopillars, which can be applied to various curved surfaces (Figure 3a). As shown in Figure 3b, the individual nanopillars have a diameter of 500 nm, a height of approximately 500 nm, and a spacing of 500 nm. The diameter of the fabricated PCL nanopillars, the height of the nanopillars, and the spacing between the columns were transferred from the PUA mold within acceptable yields.

From FT-IR spectrum of the bare PCL and nanopillared PCL (Figure 3c), we observed the following bands. The band at 2904 cm^−1^ is assigned to C-H hydroxyl group asymmetric stretching. The band at 2860 cm^−1^ is assigned to C-H hydroxyl group symmetric stretching. The band at 1722 cm^−1^ is assigned to -C=O stretching vibrations of the estercarbonyl group. The band at 1160 cm^−1^ is assigned to -C-O-C symmetric stretching [46]. The FT-IR spectra of bare PCL and nanopillared PCL had a concordance rate of more than 90%.

**Figure 4:**
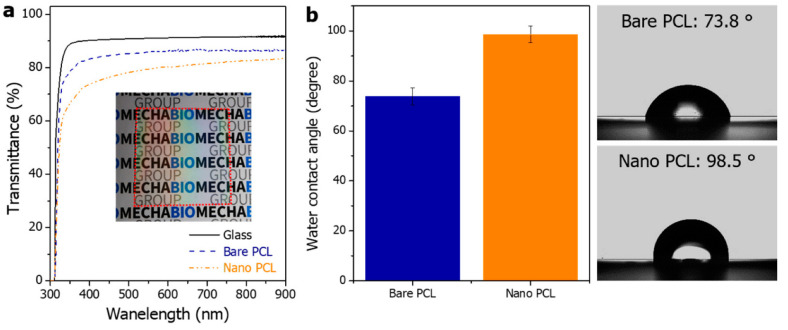
(**a**) Optical transmittance of the bare and nanopillared PCL film in the wavelength range of 300–900 nm. (**b**) Water contact angle values of the bare and nanopillared PCL surface (Nano PCL: nanopillared PCL).

Figure 4a shows the measured total transmittance spectra of glass, bare PCL and nano PCL. The bare PCL has a transmittance of approximately 85% in the visible region (visible region: 400–700 nm). The nanopillar PCL has a transmittance of approximately 75% at 400 nm wavelength and approximately 80% at 700 nm (Figure 4a). Figure 4b shows the water contact angle values of the bare PCL and nanopillared PCL surfaces. The water contact angle is influenced by a combination of the micro- and nanometer-scale roughness and the surface energy of the material [47]. The bare PCL sample had a contact angle of approximately 73.8° and the sample with the nanopillared surface had an enhanced contact angle of 98.5° due to the surface roughness. Bacterial adhesion should consider surface energy, roughness, wettability, and zeta potential. Moderate hydrophilicity and hydrophobicity may increase bacterial adhesion [48,49,50]. At the CA of 54–130°, there was a higher adsorption of bacterial peptidoglycan [51]. In addition, superhydrophobic surfaces (CA; >150 degree) have been reported to reduce bacterial adhesion because of air pockets that become entrapped between roughening features when a liquid is in contact with the solid surface. However, if entrapped air is intruded by bacterial media, the roughness then provides a larger surface area to adhere more bacteria, eventually promoting bacterial growth [48]. Therefore, it was thought that the small change in wettability shown in Figure 4b did not significantly affect bacterial adhesion. Ultimately, the bacterial adhesion of the sample requires observation between the test strain and the target surface.

### 3.2. Antibacterial Evaluation of the PCL Nanopillared Surface

**Figure 5:**
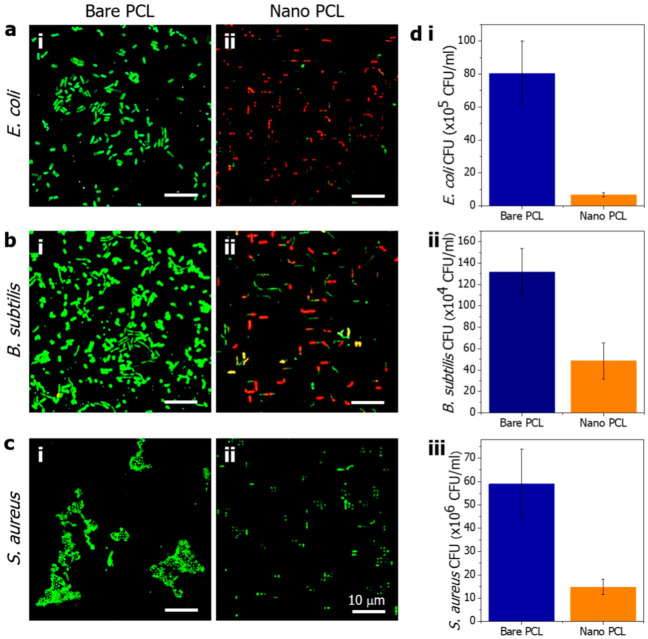
Confocal images of (**a**) *E. coli*, (**b**) *B. subtilis* and (**c**) *S. aureus* cultured on bare and nanopillared PCL samples (green: live cells, red: dead cells). (**d**) CFU of (**i**) *E. coli*, (**ii**) *B. subtilis*, and (**iii**) *S. aureus* cultured on the bare and nanopillared PCL samples for 18 h.

To evaluate the antibacterial performance of the PCL nanopillared surface, Gram-negative *E. coli* and Gram-positive *B. subtilis* and *S. aureus* bacteria were cultured on the prepared bare and nanopillared PCL surfaces at 37 °C for 18 h. Figure 5a shows confocal images of *E. coli* on the bare and nanopillared surfaces. After incubation for 18 h, live *E. coli* were adhered to the bare PCL surface, as indicated by the green fluorescence (Figure 5(ai)). However, dead *E. coli* were observed on the nanopillared PCL surface, as indicated by the red fluorescence (Figure 5(aii)). To quantitatively evaluate the bactericidal behavior of the nanopillared PCL arrays, a CFU test was performed. The antibacterial rate of *E. coli* was increased by 91.54% for the nanostructured PCL ((6.8 ± 1.3) × 10^5^ CFU/mL) compared with the bare PCL ((80.4 ± 19.7) × 10^5^ CFU/mL). This result shows that the nanopillared PCL surface has strong bactericidal effects that result from modifying the morphology of PCL to an appropriate nanopillar. The antibacterial rate of *B. subtilis* was increased by 63.24% for nanostructured PCL ((48.4 ± 16.9) × 10^4^ CFU/mL) compared with bare PCL ((131.8 ± 21.8) × 10^4^ CFU/mL), which also indicated antibacterial performance through modification of the nanopillar. The membrane damage of *B. subtilis* by nanopillars seems to have a decreased antibacterial rate compared with *E. coli* because of the thick peptidoglycan layer. In the case of *S. aureus*, the antibacterial rate was increased by 74.86% for nanopillared PCL ((14.8 ± 3.3) × 10^6^ CFU/mL) compared with bare PCL ((58.9 ± 14.9) × 10^6^ CFU/mL). Interestingly, unlike the previous two types of bacteria, *S. aureus* was alive on both bare PCL and nanopillared PCL surfaces, and the bacteria were not killed by the nanopillar. The reason for the activity of *S. aureus* on the nanopillared PCL is attributed to its relatively small size, sphere shape, nonmotile properties, and thick peptidoglycan layer. Therefore, the membrane of *S. aureus* was not damaged by the nanopillars, unlike other bacteria. However, on the nanopillared surface, *S. aureus* was stuck between nanopillars, and proliferation or activity was inhibited.

**Figure 6:**
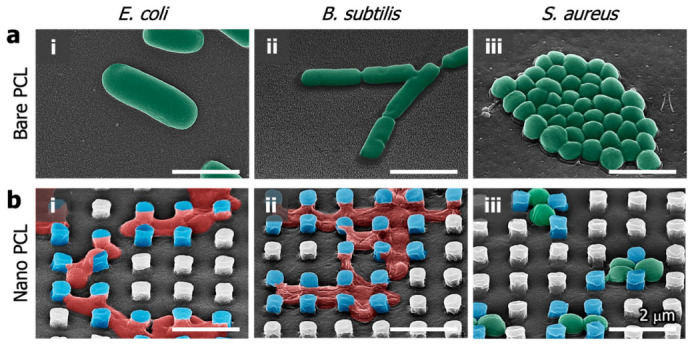
SEM images of (**i**) *E. coli*, (**ii**) *B. subtilis* and (**iii**) *S. aureus* cultured on the (**a**) bare PCL and (**b**) nanopillared PCL surfaces for 18 h.

Figure 6a shows electron microscopic images of live *E. coli*, *B. subtilis* and *S. aureus* on the bare PCL surface. On the bare PCL surface, it seems that the membranes are not damaged at all, and the bacteria in their complete form are well maintained. In the case of the nanopillared PCL surface, it seems that *E. coli* and *B. subtilis* sank down due to the membrane damage by the nanopillars (Figure 6(bi,bii)). This shows that the nanopillar array has a strong bactericidal effect, and the nanopillar PCL surface has sufficient mechanical stiffness to damage the attached bacterial membrane. *B. subtilis* and *E. coli* were attached between the nanopillars, and the bacterial membrane was attached and stretched from the top of the nanopillars to the bottom (Figure 6(bi,bii)). The damaged bacteria membrane between the nanopillars was confirmed for nonactivity through live/dead staining. As shown in the live/dead staining results of Figure 5(aii,bii), the dead bacteria have vertical (90°), diagonal (45°), and horizontal (0°) orientation. These angles are related to the nearest nanopillar direction. According to our experimental results, the mechano-bactericidal “nanopillars”are thought to damage to the bacteria membrane only by the stretching mechanism. *S. aureus* formed close-packed colonies in grape-like clusters over a large area of the surface on the bare PCL (Figure 6(aiii)). In contrast, *S. aureus* was imprisoned by a narrow space constructed by the periodic nanopillar array on the nanopillared PCL (Figure 6(biii)). It is thought that one main reason for the antibacterial activity of the periodic nanopillar array is a spatial confinement size effect which imprisons *S. aureus* between nanopillars, limits the attachment area for bacteria, and impedes the bacteria cell–cell interactions [52]. For this reason, *S. aureus* imprisoned between the nanopillars appears to be constrained in elongation and binary fission by the surrounding nanopillars. As a result, *E. coli* and *B. subtilis* were killed because the bacteria membrane was damaged by nanopillars. However, the relatively smaller *S. aureus* was attached between the nanopillars, and the proliferation was hindered. In addition, the attached *S. aureus* was all activated. Therefore, it is confirmed that *S. aureus* has a bacteriostatic effect on the nanopillar surface.

**Figure 7:**
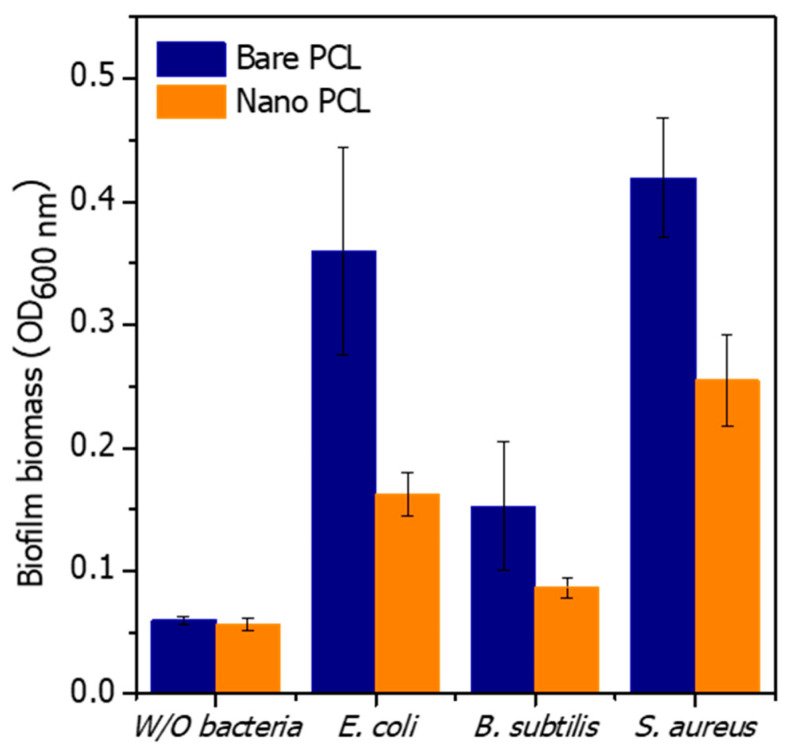
Biofilm biomass assessment on the PCL samples with crystal violet.

Bacterial biofilm biomass was quantified by CV staining (Figure 7). The OD value of CV in the PCL sample without bacteria was about 0.06 released. After 18 h of culture, the biofilm biomass of *E. coli*, *B. subtilis*, and *S. aureus* was decreased on nanopillared PCL. The biofilm biomass reduction of *E. coli*, *B. subtilis*, and *S. aureus* on the PCL nanopillars was 54%, 43%, and 39%, respectively. On nanopillared PCL, the biofilm formation of *E. coli* and *S. aureus* was significantly reduced compared with bare PCL. On the other hand, that of *B. subtilis* was slightly reduced. *B. subtilis* showed low biofilm formation on bare PCL, and the formation of biofilm was more repressed by nanopillars. The results of biofilm biomass showed a similar trend to the antibacterial results of the nanopillars (Figure 5 and Figure 7). Nanopillars can kill by damaging the membranes of the attached bacteria. In addition, bacterial growth was limited by reducing the growth area of the attached bacteria. As a result, nanopillars can inhibit the formation of biofilms on the surface.

Previously, nanopillared antibacterial surfaces were reported only in relation to membrane damage. In this study, the antibacterial ability to damage the membrane and kill the bacteria and the bacteriostatic ability to inhibit the formation of biofilms were both demonstrated. This means that the nanopillared PCL surface can use both strategies at the same time, and is not limited to one.

## 4. Conclusions

PCL is an FDA-approved material and has biocompatibility and biodegradability, so it is widely used in implantable medical devices. To use the device, it is necessary to minimize the attachment of bacteria to the surface or to kill the attached bacteria. However, devices fabricated with PCL do not have a unique antibacterial function. Therefore, firstly, we tested the mechano-bactericidal functionalization with the aid of nanopillars on the PCL surface. In this study, the process parameters for the fabrication of nanopillars were established through a thermal nanoimprinting process using PCL. Regarding the thermal nanoimprinting process, it was confirmed that a low melting point of PCL should be considered during demolding, and a demolding temperature was proposed to obtain an acceptable topology of nanopillars. We successfully fabricated nanopillars with a characteristic length of 500 nm using PCL. In addition, the PCL nanopillars were fabricated effectively over a large area (approximately 100 cm^2^) and the process was highly reproducible. The PCL-based nanopillars proposed in this study contacted the membranes of *E. coli* and *B. subtilis*, causing the bacterial membranes to stretch and damage the membranes with the strong mechanical strength of the nanopillars. A bacteriostatic effect on *S. aureus* was also demonstrated, with the bacteria being trapped between the nanopillars and its growth or activity being suppressed. The nanopillar surface showed high antibacterial performance not only against Gram-negative bacteria but also against Gram-positive bacteria, confirming that antibacterial activity is possible with PCL polymer alone. Based on the nanopillar PCL proposed in this study, it is believed that a film with high biocompatibility and mechanical strength can be used for medical devices that require an antibacterial surface.

## Data Availability

Not applicable.
